# An empirical assessment of the use of an algorithm factory for video delivery operations

**DOI:** 10.3389/frai.2024.1281110

**Published:** 2024-04-08

**Authors:** Gabor Molnar, Luís Ferreira Pires, Oscar de Boer, Vera Kovaleva

**Affiliations:** ^1^ATLAS Institute, University of Colorado, Boulder, CO, United States; ^2^Faculty of Electrical Engineering, Mathematics and Computer Science, University of Twente, Enschede, Netherlands; ^3^Divitel, Apeldoorn, Netherlands

**Keywords:** data mining, machine learning, video delivery, algorithm factory, QoS, QoE

## Abstract

**Introduction:**

Video service providers are moving from focusing on Quality of Service (QoS) to Quality of Experience (QoE) in their video networks since the users’ demand for high-quality video content is continually growing. By focusing on QoE, video service providers can provide their subscribers with a more personalized and engaging experience, which can help increase viewer satisfaction and retention. This focus shift requires not only a more sophisticated approach to network management and new tools and technologies to measure and optimize QoE in their networks but also a novel approach to video delivery operations.

**Methods:**

This paper describes the components, interactions, and relationships of an algorithm factory for video delivery operation that assures high QoE for video streaming services. The paper also showcases the results of gradually implementing an algorithm factory in the video industry. Using a dataset from 2016 to 2022, we present the case of a European PayTV service provider that achieved improved performance measured by both objective and subjective metrics.

**Results:**

The use of an algorithm factory significantly improved the PayTV service provider’s performance. The study found a fivefold increase in the speed of critical incident resolution and a 59% reduction in the number of critical incidents, all while expanding the customer base and maintaining the same level of labor resources. The case also demonstrates a strong positive relation between the productivity measures of the PayTV operator and their survey-based quality ratings. These results underscore the importance of flawless QoS and operational excellence in delivering QoE to meet the evolving demands of viewers.

**Discussion:**

The paper adds to the existing literature on relationships between operational efficiency, innovation, and subjective quality. The paper further offers empirical evidence from the PayTV industry. The insights provided are expected to benefit both traditional and over-the-top (OTT) video service providers in their quest to stay ahead in the rapidly evolving video industry. It may also translate to other service providers in similar industries committed to supporting high-quality service delivery.

## 1 Introduction

Traditional video delivery network architectures have long been the cornerstone of efficient content distribution, ensuring seamless video streaming experiences for users. However, the rapid growth of digital video consumption and the growing complexity of ecosystem components require a paradigm shift in our understanding of video delivery networks. To stay competitive, video service providers must not only provide excellent service quality but also prevent service failures. These challenges require a shift toward a comprehensive data strategy that brings together isolated data silos and harnesses the potential of advanced automation and Artificial Intelligence (AI).

Conventional approaches to network operations, which rely on siloed views, are ill-equipped to cope with the dynamic nature of today’s digital landscape (DeNapoli, 2023), and the operations of video delivery networks are no exception. Flexibility and maintainability are amongst the most relevant qualities expected from system architectures, requiring these architectures to be inherently dynamic, especially when operating in a changing environment. While traditional architectures may provide accuracy within their scope, they ultimately miss the transformative potential of harnessing data as a driving force for holistic system enhancement. Therefore, we argue that the traditional operational view of video delivery networks must be replaced by a comprehensive data-driven perspective that leverages real-time data, advanced analytics performed by Machine Learning algorithms, and continuous process improvement. By embracing this data-driven approach, operators can unlock new opportunities to optimize network performance, personalize content delivery, and enhance user experience, ultimately revolutionizing how we design, deploy, and operate video delivery networks. This paper highlights the limitations of the traditional approach, demonstrates the potential benefits of data-driven architectures, and presents practical recommendations for adopting such architectures to achieve a high level and continued QoE for the end users of video services.

QoE has been traditionally defined as “the overall acceptability of an application or service, as perceived subjectively by the end user” (ITU-T Rec. P.10, 2008: pp. 2). In later years, this definition was criticized by experts because the quality of experience is more than just meeting a certain threshold, i.e., “acceptability” is less than the “degree of delight” of the user of a service (Brunnström et al., 2013; Le Callet et al., 2013; Möller, 2017). Recognizing the need to better understand QoE, we present a practical, data-driven framework for video delivery operations that leverages existing, data-driven approaches for real-time QoE analyses and integrates it with the People, Process and Technology (PPT) framework of Leavitt (1965).

This paper introduces and outlines an algorithm factory tailored for video delivery operations, ensuring superior QoE for video streaming services. The concept of an algorithm factory refers to a systematic, automated framework designed to create algorithms that execute a series of procedural steps, which may range from relatively straightforward to highly intricate tasks. Our algorithm factory, consisting of a data-driven framework and a full service, was created specifically for PayTV service providers aiming to automate their operational processes through the use of off-the-shelf and bespoke algorithms.^1^ If implemented correctly, these algorithms will increase the PayTV operators’ automation power, i.e., their capability to independently execute a series of operations or procedures without the need for continuous human intervention. The algorithm factory allows video engineers to develop, improve, and maintain algorithms that optimize various aspects of video delivery. By continuously monitoring and fine-tuning these operational algorithms, the algorithm factory ensures that video content is delivered efficiently and reliably, with minimal quality degradation.

Paper is organized as follows: Section 2 provides a literature review. Section 3 introduces the traditional and data-driven views of video delivery networks. Section 4 showcases an early implementation of the algorithm factory, showing that data-driven methods improve productivity and that technical improvements increase customer satisfaction. Section 5 includes a discussion and describes the limitations of our approach. Section 6 gives our conclusions and suggestions for future work.

## 2 Literature review

Traditionally, the primary focus of competition amongst PayTV services has been centered around product attributes, such as screen resolution or content recommendation (Kim et al., 2017), content availability (Weeds, 2016; Katz, 2020), and service prices (Dawi et al., 2016; Azzahro et al., 2020). However, in recent years, operational efficiency and the need to transform video network operations have started getting increased attention (Patil, 2023). This increase in attention is justified by the significant disruptions and decreasing margins that the media and telecommunications fields are undergoing (Westcott et al., 2023). PayTV service providers are being forced to keep adjusting how they deliver videos to handle the growing demand, requiring at the same time both agility and impeccable service quality.

Even though many people want to watch videos online, PayTV service providers must evolve their business models by maintaining both agility and high quality (Patil, 2023). Existing models that use the same systems to send out videos for a long time are now outdated. The current viability of these systems is also increasingly compromised, entailing operational inadequacies. Therefore, the issues these companies face concern more than only adding new Internet video options. They also need to update how they charge for their services, make their services easier for people to use, ensure their services work seamlessly across different devices, and eliminate obsolete elements of their networks that are not useful anymore, so that they can keep innovating (Patil, 2023).

In addressing the challenges faced by PayTV service providers, innovation becomes a key aspect in maintaining a sustainable competitive advantage. Innovation, in a general context, involves “*making changes, large and small, radical and incremental to products, processes, and services*,” introducing something new that “*adds value to customers and contributes to the knowledge store of the organization*” (O'Sullivan and Dooley, 2009: pp. 4). In the context of this paper, innovation serves as the catalyst for PayTV service providers to address operational inefficiencies and meet evolving consumer preferences and expectations.

Despite the widespread belief that operational efficiency and innovation capabilities are crucial factors in satisfying subscribers, only limited empirical research is available that analyzes their relationship. Many studies have investigated the relationship between operational efficiency and quality (Talluri et al., 2013; Chang et al., 2017; Parast and Safari, 2022). While an increasing amount of literature is available on the relationship between innovation and quality (Gök and Peker, 2017; Kafetzopoulos et al., 2019; Rew et al., 2020), to the best of our knowledge, no empirical study has been conducted to investigate the combined relationships amongst operational efficiency, process innovation, and subjective quality *in the domain of PayTV operations*.

Service providers typically survey their customers to capture subjective measures of quality. However, these surveys are based on general population samples, are often biased, and may underrepresent the voice of detractors. Often, they are not conducted in real-time, therefore the results might be obsolete by the time they are available for decision-makers. This is one of the reasons why PayTV providers are increasingly adopting data-driven methods developed in the networking space to measure the QoE for their users (Jiang et al., 2017). These technical approaches leverage passive measurements from video application sessions to periodically update a prediction model and predict the QoE of future sessions (Jiang et al., 2016). However, these QoE predictions rely heavily on technical metrics and fail to capture a more comprehensive viewing experience.

**Figure 1:**
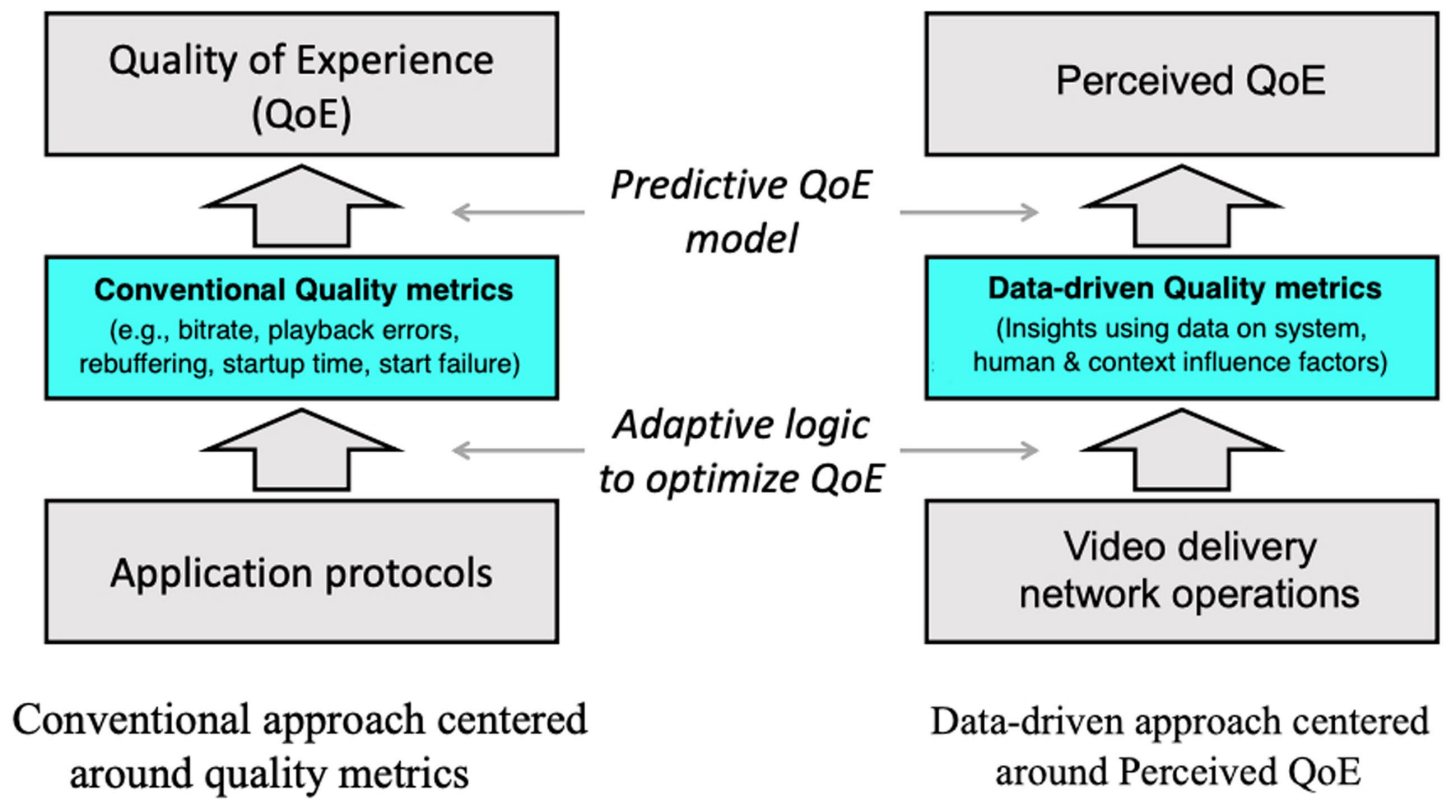
Enhancing conventional QoE assessment with data-driven QoE assessment.

The perceived QoE is not solely dependent on technical measures, but it is also influenced by multiple service aspects, including but not limited to screen resolution, content recommendation solutions, viewing options, and operational efficiency. Subscribers only perceive the video service as “delightful” if they have an overall positive experience. Therefore, to allow for a more comprehensive quality assessment, we propose a practical, data-driven framework for video delivery operations that leverages existing data-driven approaches in real-time QoE analyses (Jiang et al., 2017; Fan et al., 2018) and integrates it with the People, Process and Technology (PPT) framework that is rooted back to the seminal work of Leavitt (1965). Figure 1 shows schematically how the data-driven framework is expected to enhance QoE assessment.

Conventional QoE assessment (Figure 1) typically considers service quality metrics complemented by subjective user evaluations through surveys or interviews to gauge their perception of the service. In contrast, data-driven QoE assessment leverages advanced analytics and machine learning techniques to analyze large datasets, including data on system, human and context influence factors (Huang et al., 2018). This approach involves extracting insights from service quality data, user behavior, feedback, and operational data, providing a more dynamic and real-time understanding of QoE, often eliminating the need for explicit user input, and allowing for continuous improvement in service delivery based on data-driven insights. In addition to proposing a data-driven architecture for video delivery operations, our paper adds to the literature by empirically exploring the joint relationships between operational efficiency, innovation, and subjective quality in PayTV markets.

## 3 Video delivery operations

This section presents the traditional view of video delivery operations and our view of the data-driven platform in the context of the algorithm factory.

### 3.1 The traditional view

**Figure 2:**
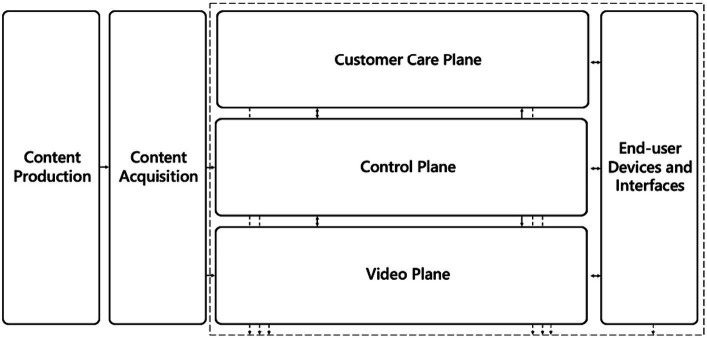
Traditional video delivery system architecture.

A traditional video delivery architecture (Figure 2) is comprised of content production, content acquisition, customer care plane, control plane, video plane, delivery network, and end-user devices. These elements work together to facilitate video content creation, acquisition, management, distribution, and consumption while ensuring a seamless and satisfying viewer experience.

The architecture for video delivery operations is quite complex and has been designed to efficiently manage and optimize the delivery of video content to end users. It consists of several interconnected components, each serving a specific purpose:

- *Content production* elements involve various tasks, such as creating, capturing, and editing video content in formats suitable for distribution. Additionally, content production includes adding metadata, subtitles, and other enhancements to enrich the viewing experience.
- *Content acquisition* elements encompass components for obtaining video content, metadata, EPG (Electronic Program Guide) information, and video advertisements from various sources. This can involve content acquisition from content creators, providers, or aggregators through licensing agreements, content ingestion, and quality assurance to ensure the obtained content meets the required standards.
- The *video plane* supports the exchange of video data packets within the network. It encompasses the video network infrastructure, including content delivery networks (CDNs) and other devices that transmit and deliver video content. It includes edge servers, caching mechanisms, and other network components that optimize the delivery process, reducing latency and improving overall streaming performance. The video plane ensures the reliable and efficient transfer of video content, following the instructions and policies set by the control plane.
- The *control plane* is at the core of the architecture. It manages conditional access, content protection, digital rights management, streaming session handling, load balancing, traffic management, Quality of Service (QoS) control, and resource allocation. The control plane ensures accurate and efficient content distribution.
- The *customer care plane* provides support and assistance to end users. This component handles customer service operations, addressing inquiries, resolving issues, and managing customer accounts. By ensuring timely support and addressing end-user concerns, the customer care plane contributes to a positive user experience.
- Viewers use end-user devices and interfaces to access and consume video content. These devices can vary from smartphones, tablets, and computers to smart TVs, set-top boxes, and other connected devices. End-user devices provide the interface for users to interact with the video delivery system, enabling them to browse, select, and play video content.

Collectively, these components represent the architecture for video delivery operations. They ensure efficient content production, seamless acquisition and delivery of video content, responsive customer support, and optimal network management. This comprehensive system supports the overall video streaming experience, providing viewers with high-quality content and a user-friendly interface across various devices. This traditional architecture possesses technical accuracy but lacks a comprehensive data-driven operational view of the video delivery system. While this legacy approach may be suitable in static scenarios, it lacks automated capabilities for advanced monitoring and dynamic improvements.

### 3.2 A data-oriented view: the algorithm factory

**Figure 3:**
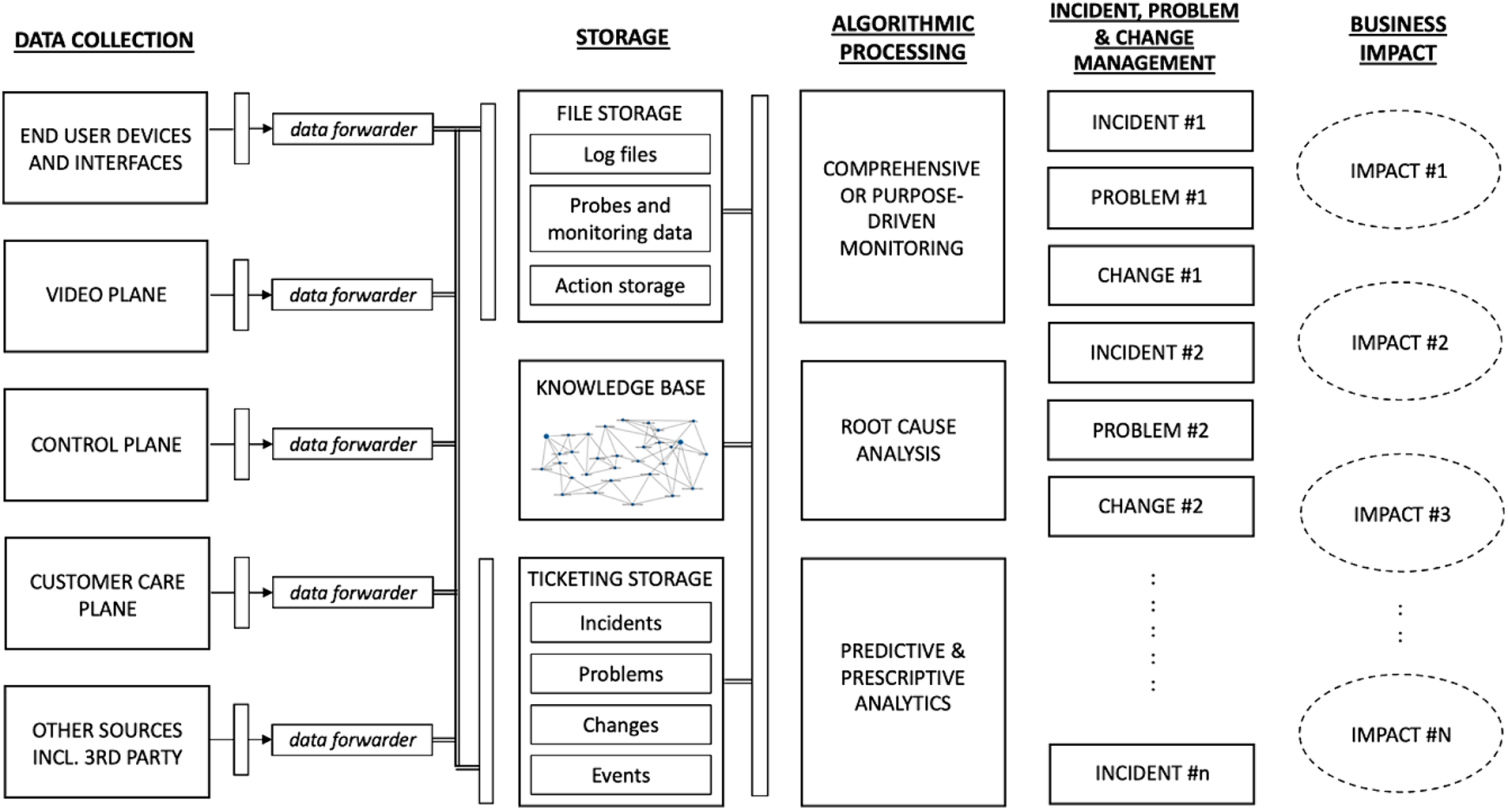
Algorithm factory for video delivery network operations.

The algorithm factory for video delivery operations (Figure 3) is a data-driven framework that focuses on developing, optimizing, and deploying algorithms specifically designed for enhanced video delivery. It is a facility where various algorithms and techniques can be designed, refined, and implemented to enhance the video delivery process and improve the overall user experience. The algorithm factory may use real-time and historical data from various sources, including end-user devices, video plane, customer care plane, and (if relevant) other third-party sources (e.g., social media data or weather information).

Key capabilities of the algorithm factory for video delivery include:

- *Algorithm development*: The factory serves as a hub for the research, development, and innovation of algorithms tailored for video delivery operations. It involves designing algorithms that optimize all aspects of video transmission.
- *Optimization and testing*: The algorithms created in the factory undergo rigorous testing and optimization processes. This involves assessing their performance under different network conditions, user scenarios, and device capabilities. The goal is to refine and fine-tune the algorithms for efficient video delivery, minimizing buffering, reducing latency, and ensuring smooth playback. Well-defined procedures perform optimization and testing to guarantee the stability of the system and the services offered. We use a release train for client and frontend releases where each client firmware release and frontend deployment are always first tested in the live network with an *alpha* and a (larger) *beta* group of users. Both groups are actual customers in a real household environment, and they provide structured feedback about their experience by responding to a survey every two weeks. When no issues are found in the first round of testing with the *alpha* group, and the feedback is positive, the same release is deployed to the *beta* group. If this deployment is successful, the new release is deployed to the entire customer base. For backend and system releases, all operational changes to the production platform are first tested on a pre-production system. The pre-production platform is similar to the production system but has only a subset of the hardware, software, and infrastructure and has a limited number of test users. For a stress test, we simulate a fictive load on the system to stress the resources and measure its performance under these load conditions.
- *Algorithm selection and integration*: The factory identifies the most effective algorithms and integration approaches for specific video delivery scenarios. It considers factors such as available network bandwidth, user device types, content characteristics, and user preferences. The selected algorithms are integrated into the video delivery system to enhance its capabilities.
- *Continuous improvement*: The factory is a dynamic environment where ongoing research and development are constantly working in the background. It focuses on continuous improvement, incorporating feedback, monitoring performance metrics, and adapting algorithms to evolving network conditions, content formats, and managing user expectations.

Additionally, the factory enables and encourages collaboration between content creators, streaming platforms, content delivery networks (CDNs), and other stakeholders in the video delivery ecosystem. These partnerships can facilitate the exchange of knowledge, data, and insights, leading to advancements in algorithm design and implementation. The algorithm factory goes beyond simply creating a data-driven information plane, as it entails the establishment of a comprehensive and adaptable framework that not only leverages data-driven insights but also integrates complex algorithmic processes. The algorithm factory encapsulates the entire lifecycle of algorithmic development, from design and testing to deployment and continuous refinement. This approach acknowledges the dynamic nature of video networks, requiring algorithms that respond to real-time data and learn from it to evolve and self-improve over time. By encompassing this broader scope, an algorithm factory acknowledges the intricate interplay between data and algorithms, fostering a symbiotic relationship that enables video networks to operate efficiently, adapt and thrive amidst the ever-evolving landscape of network demands and technological advancements.

### 3.3 Evolution of the operational architecture

The PayTV industry is often characterized by rapid technological advancement, the traditional static and rigid system architecture is becoming increasingly outdated and ineffective. It was gradually replaced by a more data-driven architecture designed in accordance with the Algorithm Factory view. Table 1 highlights the evolution of the Algorithm Factory. It has been an ongoing process driven by the dynamic nature of our industry, technological advancements, and the changing business needs of the service provider.

**Table 1:** Evolution of the algorithm factory.

Phase	Years	Primary focus	Notes
Conceptualization	2016–2018	Design and planning, initial data collection	Platform development and service launch.
Data capture	2019–2020	Comprehensive data collection	General availability (GA) of service; large increase in the number of customers.
Automation	2021–2022	Algorithms and refinements (ongoing)	Increased productivity and operational control.

### 3.4 People, process, and technology

Operating an algorithm factory for video operations requires aligning people, technology, and processes. While advanced algorithms form the core of operations, the human element remains indispensable for refining, validating, and enhancing these algorithms. Skilled engineers and data scientists are vital for creating and fine-tuning the algorithms, ensuring they align with specific video processing goals and adapt to evolving needs. Technology is the backbone, providing the infrastructure for algorithm development, data storage, and computation. Cutting-edge hardware accelerates processing speeds, enabling real-time video analysis. However, well-defined processes orchestrate these components into a cohesive workflow, from data collection and preprocessing to algorithm training and deployment. Systematic processes streamline operations, enhance collaboration between cross-functional teams, and guarantee the delivery of high-quality video experience. Ultimately, the synergy between people, technology, and processes forms the cornerstone of an effective algorithm factory for video operations, yielding innovation and efficiency in equal measure. In fact, the “algorithm” in the algorithm factory can be more broadly defined as the (automated or manual) procedure and process to be applied to a set of inputs to create a desired output and reach a goal. An algorithm can be implemented solely in computational software. However, it can alternatively include creating, updating, running, and maintaining this software, interpreting its results, and acting on its outcomes.

Leavitt’s PPT framework (Leavitt, 1965) is widely recognized in operational management and improvement initiatives. It emphasizes the crucial role of people, processes, and technology in achieving successful organizational performance and efficiency.^2^ The synergy and alignment of these elements are essential for its success. Often, senior management tends to invest heavily in cutting-edge technology and recruiting and training top-tier employees (Kiron et al., 2020). While this strategy is essential, it may not yield the desired outcomes. Investing in state-of-the-art (e.g., cloud) infrastructures, innovative applications, and advanced monitoring tools is commendable, but does not guaranteed success. This principle also applies to staff recruitment. Significant time and effort are spent in finding the right talents to learn and operate these new technologies, with often disappointing results. At the same time, the current workforce requires proper retraining to adapt to evolving technological demands. The ever-competitive labor market necessitates broadcasting and telecom industries to compete with various sectors for skilled IT engineers and data scientists, which makes the challenge even bigger.

## 4 Evidence from an algorithm factory

This section discusses our empirical research using data from an Algorithm Factory of Divitel, a professional services company based in the Netherlands. We present the case of a European PayTV service provider and analyze a unique dataset from 2016 to 2022, including technical, operational, and customer satisfaction data collected from multiple sources. The mid-size service provider owns its own Hybrid Fiber Coax (HFC) and Fiber-to-the-Home (FttH) infrastructure. It also offers television services over the top to customers beyond its network. Its TV platform is geo-redundant and deployed fully on-premises. This PayTV service provider operates in one of Europe’s most digitally sophisticated and innovative markets, where consumers demand high-quality products and services. The consumer market in this European country is known for its high purchasing power, reflecting a prosperous and stable economy.

Our analysis uses a comprehensive data set extracted from an ITIL-based (Cartlidge et al., 2021) trouble ticketing system, which captured seven years of historical data on incidents, problems, events, and changes. The data set also provided information about the Average Resolution Time for incidents, changes, and problems and their severity levels.^3^ In the ITIL methodology, *incidents* refer to any unplanned interruption or reduction in the quality of service, a *problem* is the underlying cause of one or more incidents, *changes* are any modifications to the existing IT infrastructure or introduction of a new one, and an *event* is any significant occurrence that requires attention, such as the failure of a device or the detection of a security breach (Brenner, 2006). By using the ITIL framework, PayTV operators can effectively manage incidents, problems, changes, and events to ensure optimal performance and customer satisfaction.

Technical and operational data were collected from the company’s internal trouble ticketing system (Jira^4^) from February 27 till March 3, 2023. For data cleaning, we sorted all the incidents per provider, selected the provider of our study, and then sorted the incidents by year, incident type, and severity level, focusing on trouble tickets with critical severity level. To perform our analysis, we combined the trouble ticketing system data with subjective, survey-based assessments collected directly from subscribers by an independent third party.^5^ These assessments included total TV service quality rating, quality score, innovation capability, and customer support and covered the same period as our data set. Tables 2, 3 describe the key variables of our study.

**Table 2:** The list and description of the key variables of the study.

Variable	Description
No_of subscribers	The number of TV subscribers for the TV operator.
No_of_incidents	Incident is an unplanned interruption to the service or a reduction in the quality of a service. It can also be an event that has not yet affected the service but could cause an adverse impact. This variable shows the number of Incidents within a given time period.
No_of_problems	The root cause of one or more incidents. Problem management is the process that manages the lifecycle of all problems. This variable shows the number of Problems within a given time period.
No_of_changes	Any alteration or modification to a system or service is considered a change, including the addition of new services. ITIL defines a change as the addition, modification, or removal of anything that could have an effect on the services. This variable shows the number of Changes within a given time period.
No_of_events	This variable shows the number of events within a given time period, indicating the depth and breadth of monitoring
Quality ratings	
Total TV Service Quality Rating	The Total TV Service Quality rating is calculated by assessing the TV Platform performance by its Innovation, Flexibility, Quality, Price, and Customer Service Quality scores.
TV Service Quality Score	Survey-based quality rating to show how well the quality of the TV service meets or exceeds end users’ expectations.
Innovation Score	Innovation score to measure how quickly new services/features are made available to end users.
Customer Support Score	Customer Support score to the quality of the service desk (the competence of the helpdesk, the quality of their advice, and helpdesk availability).

**Table 3:** Incident types and their descriptions.

Incident types	Description
Boot process	An incident related to the boot process might involve issues with the start of a device, such as a set-top box or TV, such as the device not starting up at all, or taking a long time to start.
Audio selection	An incident related to audio selection might involve problems with selecting the desired audio track for a video, such as the audio being out of sync, or not playing at all.
Channels—favourite channels lists	An incident related to favourite channel lists might involve issues with the creation, management, or access of the customer’s favourite channel list. For example, the list might not be saved correctly, or the customer may not be able to access it.
Channels—locked channels	An incident related to locked channels might involve issues with channels that have been locked, such as the customer not being able to unlock them, or the channels being locked without the customer’s consent.
CPE & RCU (remote control unit)	An incident related to Customer Premises Equipment (CPE) and Remote Control Units (RCU) might involve issues with the device itself, such as hardware failures or firmware updates, or issues with the remote control, such as the device not responding to the remote or the remote not working at all.
CPE Provisioning and SW Download	An incident related to CPE provisioning and software download might involve issues with the customer’s device not being properly provisioned or updated, such as firmware updates failing or not completing successfully.
EPG (Electronic Program Guide)	An incident related to the Electronic Program Guide might involve issues with accessing or navigating the guide, such as the guide not displaying correctly, or the customer not being able to find the desired program.
General TV & Zapping (surfing)	An incident related to general TV and zapping might involve issues with viewing and navigating TV channels, such as channels not displaying correctly or the customer experiencing problems while changing channels.

With exploratory data analysis, we aimed to understand the relationships between key variables: the number of subscribers, TV service quality scores, incidents, problems, changes, and events, as well as the duration of solving incidents and the time needed to implement changes.

### 4.1 Incidents resolution speed

**Figure 4:**
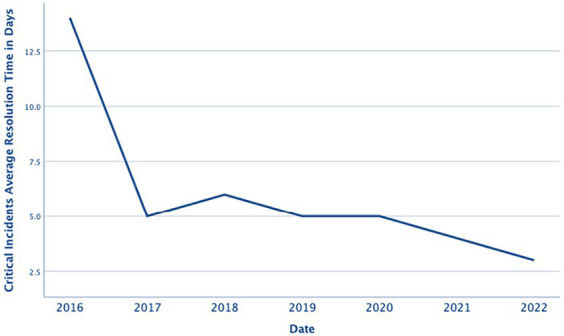
Average resolution time to repair critical incidents (in days).

The average time required to solve critical incidents in the period from 2016 to 2022 is presented in Figure 4. The figure shows that the troubleshooting efficiency has significantly increased, as evidenced by the decreasing average time required to solve critical incidents. The decrease in average resolution time to solve critical incidents is significant, with a reduction of 79% (from 14 days in 2016 to 3 days in 2022). This translates to incidents being now solved about five times faster than before.

### 4.2 Number of incidents and problems

**Figure 5:**
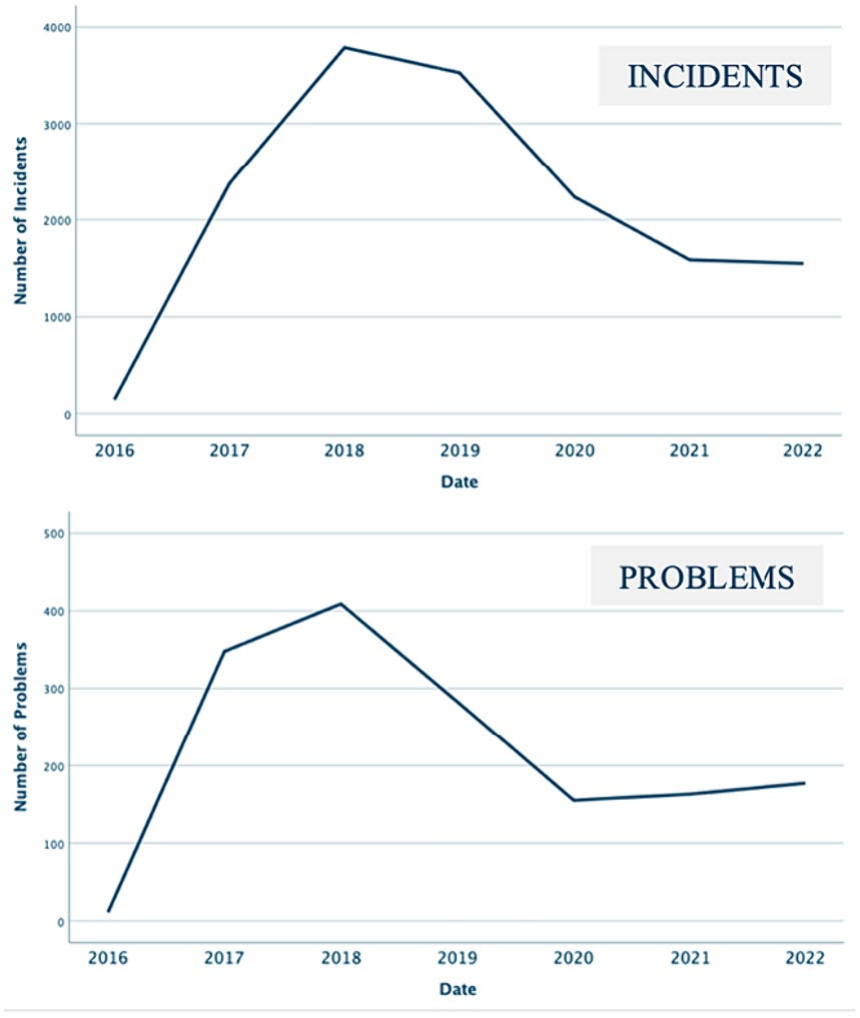
The number of incident tickets and problems in each year.

The number of incidents and problems from 2016 to 2020 are presented in Figure 5. Both the upper and the lower graphs suggest a significant improvement in the stability of the Pay TV platform since the video service was made widely available in 2019. Specifically, there has been a 59% decrease in the number of incident tickets reported (down from 3,786 in 2018 to 1,548 in 2022) and a 57% decrease in the reported problems (down from 409 in 2018 to 177 in 2022). These decreases suggest that the TV service delivery platform has become more dependable and stable. However, we observe that during the initial years, the number of incidents increased considerably and peaked at 3,786 in 2018, while the number of problems increased to 409 in 2018. This increase shows the value of implementing a data-driven approach. As a result, the system’s monitoring capabilities increased, and better situational awareness enabled the monitoring system to detect and log more incidents than before.

**Figure 6:**
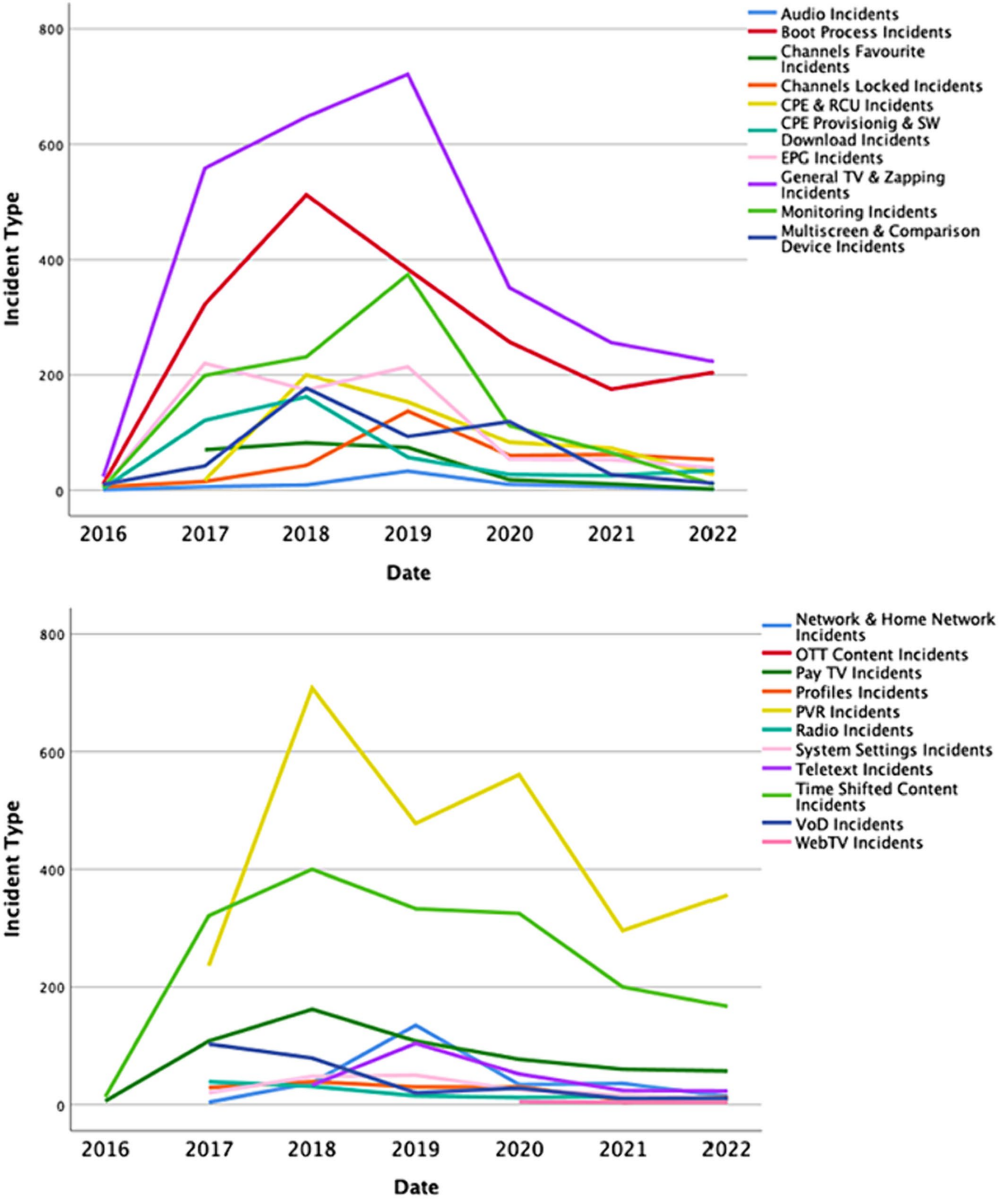
The number of incident types.

The changes in the number of incidents, categorized according to the major incident types, are highlighted in Figure 6. The figure indicates a similar trend to Figure 5, indicating that the number of incidents decreased not just for the total number of incidents but also for each incident type.

**Figure 7:**
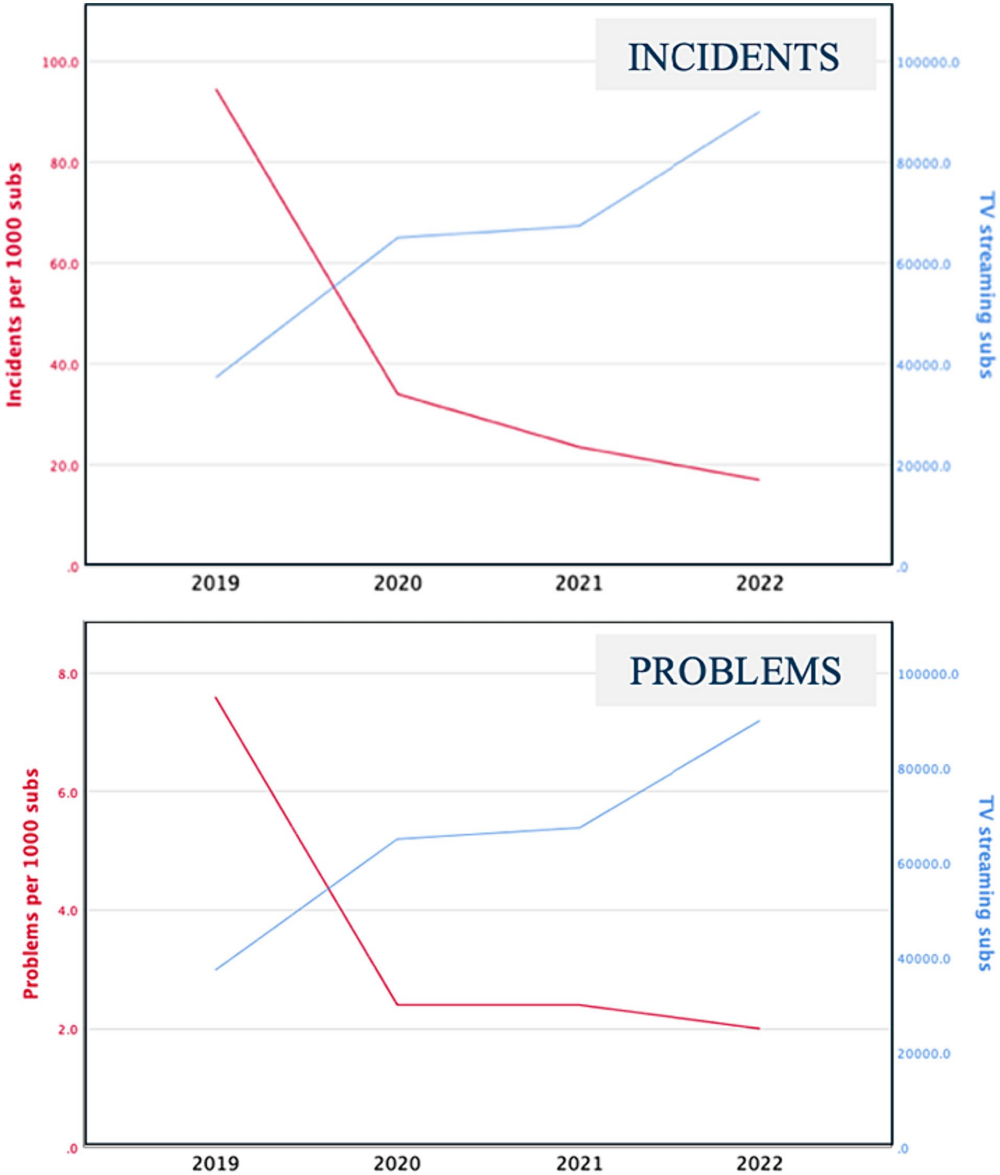
Number of TV subscribers and problems per 1,000 subs.

We found that there has been an 82% reduction in incidents per 1,000 subscribers, with the number dropping from 94.5 to 17 (Figure 7). This means that in 2019 there were 94.5 incidents per 1,000 subscribers, but by 2022, this number had decreased to 17. In other words, there are now only one-fifth as many incidents per 1,000 subscribers as before, which represents a reduction by a factor of 5. Figure 7 also shows that the number of problems per 1,000 subscribers decreased from 7.6 to 2 from 2019 to 2022. This means there were four times fewer problems per 1,000 subscribers in 2022 than in 2019.

### 4.3 Changes and implementation lead time

In addition to solving incidents and problems, the PayTV service provider also implemented changes in the system to ensure that the services stayed aligned with the needs of the business. Changes may be made in response to problems that have been identified to improve service performance or availability, implement new functionality, or address known or potential risks.^6^ The change process involves a series of steps that are designed to minimize the risk of disruption to the video services while implementing the change. This includes a formal change request process, impact and risk assessment, testing, approval, and implementation planning.

**Figure 8:**
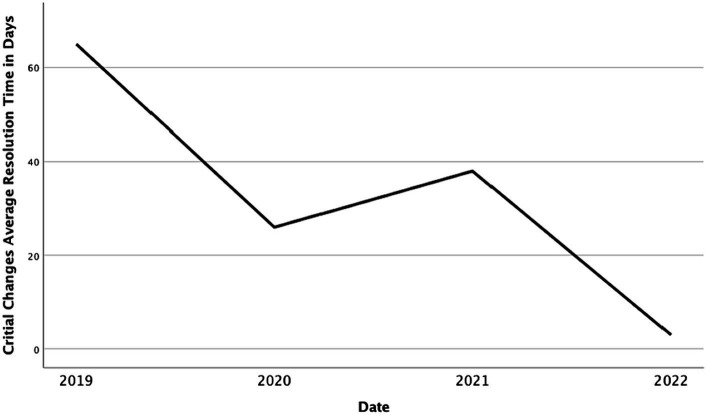
Average number of days needed to implement critical changes.

The lead times to implement critical changes from 2019 to 2022 in terms of the average number of days necessary to implement these changes are presented in Figure 8. The figure indicates that this time has also decreased by 22 times since the video service was made widely available in 2019 (down from 65 days in 2019 to 3 days in 2022).

**Figure 9:**
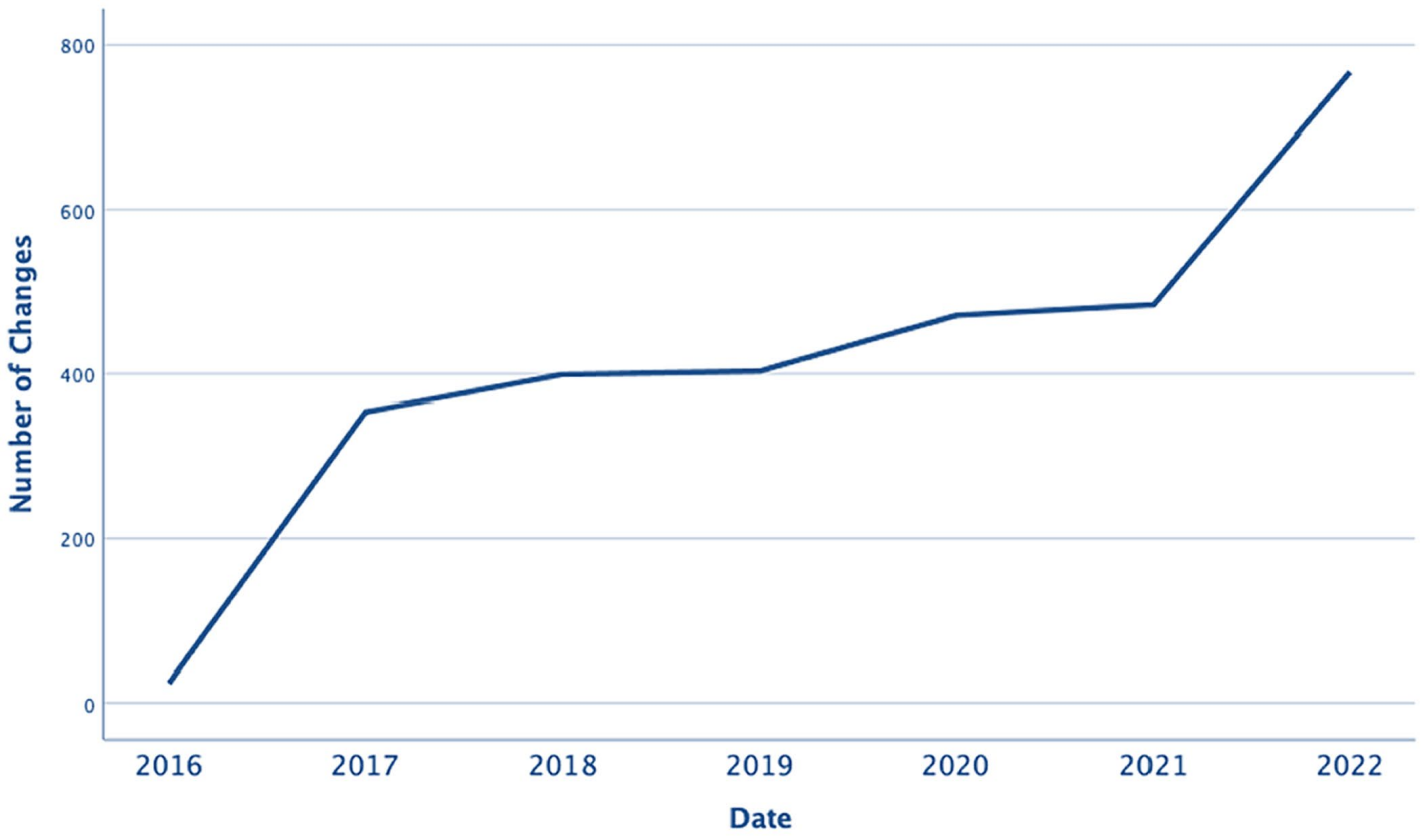
Number of changes in each year.

Figure 9 shows the number of changes that occurred each year between 2016 and 2022, indicating that the number of changes increased by 216%. In 2017, 354 changes were implemented, while in 2022, the number of changes rose to 767.

### 4.4 Correlational analysis

The exploratory data analysis showed that incident resolution speed and the lead time to implement changes had improved significantly, resulting in fewer incident tickets and the capability to implement more changes. These positive developments suggest that the TV platform has become more stable and reliable, showing that adapting to subscribers’ evolving needs and taking a data-driven approach to operations leads to improved productivity. However, we had to investigate variable relationships to determine if a correlation exists between enhanced productivity and higher subscriber satisfaction.

We employed Pearson’s correlation coefficient (Freedman et al., 2010) to measure the strength of the relationship between productivity measures and subscriber satisfaction indicators. This method calculates the correlation coefficient, which quantifies the degree to which two variables tend to change together. A correlation score of 1 indicates a perfect positive linear relationship, while 0 implies that there is no linear relationship. The significance of the correlations was tested using a two-tailed test, appropriate for our study as it does not assume a direction of the relationship. The observation period, denoted by N, refers to the number of years over which data was collected, providing a longitudinal perspective to our analysis. To further validate our findings, the *p*-values were assessed to determine the statistical significance of the results. A *p*-value smaller than 0.05 indicates that the observed correlation is highly unlikely to have occurred by chance, hence suggesting a true relationship between the variables under consideration.

Table 4 shows the correlation scores between selected productivity measures and the TV quality and innovation scores, respectively, quantifying the strength and direction of the linear relationship between any two variables. We found a moderately strong positive correlation between the number of changes and the total TV Service Quality Rating (= 0.721) and the innovation score (=0.703), which implies that there is a positive relationship between the number of changes and the TV platform’s quality scores, suggesting that the overall system quality was continuously improved with the changes. The correlation table (Table 4) identifies a strong and significant positive correlation between the number of events and the total TV Service Quality Rating (= 0.822) and the innovation score (=0.758) suggesting the depth and breadth of monitoring positively impact both quality rating and the innovation score.

**Table 4:** Correlations between productivity and subscriber satisfaction.

		Number of changes	Number of incidents	Number of problems	Number of events	Total TV service quality rating	Innovation score
Number of changes	Pearson correlation	1	0.294	0.267	0.713	0.721	0.703
	Sig. (2-tailed)		0.523	0.562	0.072	0.067	0.078
	*N*	7	7	7	7	7	7
Number of incidents	Pearson correlation	0.294	1	0.889^**^	−0.218	−0.377	−0.358
	Sig. (2-tailed)	0.523		0.008	0.639	0.404	0.430
	*N*	7	7	7	7	7	7
Number of problems	Pearson correlation	0.267	0.889^**^	1	−0.373	−0.408	−0.424
	Sig. (2-tailed)	0.562	0.008		0.410	0.364	0.343
	*N*	7	7	7	7	7	7
Number of events	Pearson correlation	0.713	−0.218	−0.373	1	0.822^*^	0.758^*^
	Sig. (2-tailed)	0.072	0.639	0.410		0.023	0.048
	*N*	7	7	7	7	7	7
Total score in Bilanz TV rating	Pearson correlation	0.721	−0.377	−0.408	0.822^*^	1	0.954^**^
	Sig. (2-tailed)	0.067	0.404	0.364	0.023		0.001
	*N*	7	7	7	7	7	7
Innovation score in Bilanz TV rating	Pearson correlation	0.703	−0.358	−0.424	0.758^*^	0.954^**^	1
	Sig. (2-tailed)	0.078	0.430	0.343	0.048	0.001	
	*N*	7	7	7	7	7	7

^**^Correlation is significant at the 0.01 level (2-tailed).

^*^Correlation is significant at the 0.05 level (2-tailed).

### 4.5 Interpretation

Our analysis of the empirical data has shown that the automation power of the PayTV service provider increased significantly, resulting in notable enhancements to its operational efficiency. Automation power in this context refers to a PayTV operator’s ability to automate routine and repetitive tasks associated with operating their video delivery platform and providing services to subscribers. These tasks include monitoring platform performance, deploying software updates, managing backups, and handling incidents and changes. Other operational efficiency factors, such as process optimization, resource management, performance management, continuous improvement, and risk management, are also essential.

The study provides empirical evidence for the following:

- A decrease in the time it takes to resolve incidents.
- A substantial decrease in the number of incident and problem tickets, despite the growing number of customers and increasing complexity of the system.
- A notable increase in the capability of the PayTV operator to implement changes in operations, with practically the same workforce.

Our findings also suggest that more data-driven monitoring leads not just to higher efficiency in solving incidents and problems but also increases the PayTV service provider’s capability to implement more changes and be more innovative. By resolving incidents more rapidly, the operator has more time and resources for making changes and adding new features with the same amount of labor resources. This implies that data-driven problem resolution and automating tasks can increase the accuracy and consistency of services provided by PayTV operators, promote innovation, and improve overall customer satisfaction.

## 5 Discussion

Section 3 described the components, interactions, and relationships of an algorithm factory for video delivery operation. We argued that such an algorithm factory allows video engineers to develop, improve, and maintain algorithms that optimize various aspects of video delivery. Using a dataset from 2016 to 2022, in Section 4, we presented the case of a European PayTV service provider that achieved improved performance measured by objective and subjective metrics. In this section, we emphasize the importance of people and processes.

### 5.1 Operational processes

In today’s rapidly evolving video delivery landscape, the choice of technology has become widely available, with numerous new upcoming vendors offering innovative products (Westcott et al., 2023). However, despite adopting these advanced tools to address operational challenges, they are often underutilized due to resource constraints. This concerns not only the quantitative number of resources but, more frequently, not the right qualitative skill set and competencies that can be found or trained to be assigned to the job. After installation and delivery by the vendor, the operational team has difficulties adapting and learning the new technology and may revert to old habits, unable to fully grasp and harness the latest technology’s potential. On top of that, these technologies typically come with high operation (OPEX) costs. In combination with the limited incremental value it adds to the business, the technology often does not remain in use for long. It is then blamed for the failure, and the senior management pursues the next best technical solution, perpetuating a repetitive cycle but just with a different vendor.

The *process* aspect of the PPT model is a critical component often overlooked, yet it plays a pivotal role in driving operational and organizational success. Processes are more than mere connectors between technology and people; they serve as enablers, fostering a robust ecosystem of productivity and effectiveness. However, when processes are poorly implemented, they can become obstacles instead of accelerators for great people using great tools. Ineffectively designed processes create unnecessary overhead, bureaucracy, and frustration, ultimately hindering business growth and discouraging proactive improvement. In addition, we acknowledge that we are not dealing with isolated, simple processes but rather a series of iterative actions, steps, or operations performed in a specific sequence to achieve a particular result or goal. Ensuring alignment amongst these processes and reducing manual handover points is vital for success, as failure becomes more likely when misalignments and inefficiencies are present. Therefore, a more thoughtful and sustainable approach is needed than just implementing another process. Algorithms can improve operational processes by bringing automation, standardization, optimization, scalability, predictability, and data-driven decision-making to a standard process. Implementing well-designed algorithms can increase efficiency, reduce costs, and enhance productivity, driving operational success. An algorithm is not necessarily better than a standard operational process; instead, it is a tool that can improve and optimize the day-to-day processes when used appropriately.

Moreover, algorithms should not be viewed as standalone solutions but as part of a broader digital transformation strategy. Organizations must invest in adequate training and skill development to equip their workforce with the necessary knowledge to work alongside algorithms effectively. Balancing algorithms’ strengths with human insights and creativity can lead to a more agile and adaptive operational environment. Furthermore, continuous monitoring and evaluation are crucial to measure the impact of algorithms on operational processes. Organizations can identify areas for further optimization and fine-tuning of the algorithms by collecting and analyzing relevant data, ultimately driving ongoing improvements. It is crucial to consciously design, develop, and implement the appropriate algorithms to improve operational processes, but this should not be seen as the sole end goal. The actual objective is to foster a seamless synergy between people and technology, creating a harmonious collaboration that drives organizational success. By strategically incorporating algorithms, organizations can achieve better, more automated, and more efficient processes, empowering their workforce to work best with the newest evolving technologies.

### 5.2 Research limitations

The empirical part of our paper focuses on a single case study of a European PayTV service provider, which limits the generalizability of the findings to other video service providers and contexts. The specific characteristics, infrastructure, and operational challenges of this service provider are expected to be representative for the broader video delivery industry, but this might not be the case. We also acknowledge that our simple analysis does not formally establish causal relationships between operational efficiency, innovation, and subjective quality. Therefore, the limitations of this study impact its applicability in two ways. First, the findings may not universally apply to diverse video service providers with distinct practices and technological contexts. Second, the correlational nature of the analysis limits our ability to draw definitive conclusions about the causal impact on various performance metrics and customer satisfaction, but indicates that a relationship probably exists.

Despite the known limitations of exploratory data analyses^7^ and correlational research studies,^8^ and the single case study, we believe that the results provide a more nuanced understanding of the possible causal relationship between operations excellence, innovation, and service quality in the digital TV industry, helps identify patterns and relationships between variables that may not be immediately apparent, and contribute to the development of new best practices within the PayTV industry. The identified limitations also present opportunities for future research, encouraging endeavors such as comparative studies across diverse providers, exploration of additional variables, and elaborating experimental designs to bolster the evidence base and enhance the generalizability of our findings across the broader video service provider landscape.

While our case study provides promising insights into the effectiveness of an algorithm factory within our industry, contextual factors that might limit the transferability of our results need to be considered. Different regulatory environments, market dynamics, and consumer behaviors across regions may significantly influence the efficacy of algorithmic solutions in video delivery operations. For instance, varying levels of technological infrastructure, such as available network bandwidth and data processing capabilities, could result in differential outcomes when implementing the same algorithmic approaches. Service providers with different scales, ranging from multinational corporations to local providers, may also experience varied impacts due to differences in their operational complexity and resource availability. In addition, cultural factors could affect user interface preferences and content personalization algorithms, requiring adaptations to the algorithm factory framework to cater to local tastes and expectations. Future research should explore these variations by implementing the algorithm factory concept in diverse contexts and studying its outcomes across different service providers. Methodologies such as controlled experiments could provide more substantial evidence of causality between the use of algorithm factories and performance improvements. Key variables for future studies might include algorithm adaptation rates, cost–benefit analyses comparing manual and automated processes, and the effectiveness of algorithms in real-time incident resolution. It is also essential to develop more comprehensive data-driven approaches for measuring the qualitative aspects of video delivery, such as content relevance and viewing experience. This should enable a more nuanced understanding of where and how algorithmic optimization can be most beneficial and identify contexts where traditional methods may still hold an advantage. By expanding the scope of investigation beyond a single-case study, we can better determine the broader applicability of the algorithm factory and provide a more globally comprehensive set of recommendations for the video delivery industry.

## 6 Conclusion

Innovation and operation are contradictory by nature. On the one hand, video service providers need to innovate to stay competitive, and their innovation activities involve developing new features, adopting cutting-edge technologies, or offering new services to attract and retain users. On the other hand, operational stability is crucial for the day-to-day functioning of their service delivery platform. Providers must operate a reliable streaming infrastructure, manage the overall efficiency of the platform, and offer excellent customer support services. The contradiction arises because the pursuit of innovation often involves taking risks, experimenting with new ideas, and making changes that can disrupt the smooth operation of the service.

This paper introduced a data-driven framework for video delivery operations that can leverage advanced analytics and machine learning techniques. While effective in the past, the traditional view of video delivery networks had to evolve to address the challenges posed by the rapid growth of digital video consumption and the increasing complexity of ecosystem components. We proposed the concept of an algorithm factory as a data-oriented framework for video delivery operations. The algorithm factory focuses on developing, optimizing, and deploying algorithms specifically designed for video delivery. It enables continuous improvement and collaboration amongst stakeholders in the video delivery ecosystem, facilitating algorithm design and implementation advancements.

Through empirical research using data from an early implementation of an algorithm factory, we demonstrated the benefits of adopting a data-driven approach in video delivery operations. Our analysis showed a significant increase in operational efficiency, as evidenced by a decrease in the time required to resolve critical incidents and a substantial reduction in the number of incident and problem tickets. These improvements were achieved despite the growing number of customers and the system’s increasing complexity.

The empirical evidence presented in this paper demonstrates the potential benefits of adopting a data-driven view, providing a foundation for future research and practical implementations in video delivery networks. Empirical research in this domain is scarce, and the insights provided by our empirical assessment can greatly benefit service providers in their quest to stay ahead in the rapidly evolving PayTV industry. The empirical evidence also underscores the transformative potential of a data-driven framework in reshaping video delivery operations. The contradiction between innovation and operational stability is a common challenge for video service providers, and the demonstrated benefits of the automation power have broader implications for the future design, deployment, and operation of video delivery networks on a larger scale. The introduction of the algorithm factory as a dedicated framework for developing, optimizing, and deploying algorithms tailored for video delivery operation represents a departure from traditional views of video delivery networks. It also opens avenues for continuous improvement and collaboration amongst stakeholders in the video delivery ecosystem.

## Data availability statement

Publicly available datasets were analyzed in this study. This data can be found at: https://doi.org/10.17605/OSF.IO/KR83S.

## Author contributions

GM: Writing – original draft, Writing – review & editing. FL: Writing – original draft, Writing – review & editing. OB: Writing – original draft, Writing – review & editing. VK: Writing – review & editing, Data Curation, Visualization.

## Footnotes

^1^In this context, an algorithm refers to a “finite, abstract, effective, compound control structure, imperatively given, accomplishing a given purpose under given provisions” (Hill, 2016: pp. 47).

^2^Over the last few years, there have been increasing arguments for adding “Data” to the framework (e.g., White, 2017; Gold et al., 2023).

^3^The trouble ticketing system recognizes four severity levels: critical, high-priority, normal, and low-priority. Our analysis focused on tickets with critical severity levels, using data from 2016 to 2022. We studied a period of seven years during which technological and process improvements were implemented.

^4^ https://www.atlassian.com/software/jira

^5^These survey-based assessments are part of an independent third party’s yearly evaluation of PayTV service providers in the local market. The yearly evaluation aims to provide end consumers with an objective and reliable comparison of TV Service Quality measures to help them make informed decisions. The quality assessments are widely recognized and considered essential benchmarks. We used data from the assessments done in the years 2016–2022.

^6^The reason for the changes can be, for example, to cope with the growing number of subscribers and to extend the CDN (Content Delivery Network) or Middleware frontends.

^7^For details on the limitations of exploratory data analysis, see Chatfield (1986).

^8^For details on the limitations of correlational research methodology, see Mitchell (1985).
